# Characteristics of Dietary Fatty Acids Isolated from Historic Dental Calculus of the 17th- and 18th-Century Inhabitants of the Subcarpathian Region (Poland)

**DOI:** 10.3390/molecules26102951

**Published:** 2021-05-15

**Authors:** Joanna Rogóż, Magdalena Podbielska, Ewa Szpyrka, Maciej Wnuk

**Affiliations:** 1 Institute of Archaeology, University of Rzeszow, Aleja Rejtana 16c, 35-959 Rzeszow, Poland; rogoz.joanna@gmail.com; 2 Department of Biotechnology, University of Rzeszow, Aleja Rejtana 16c, 35-959 Rzeszow, Poland; magdapodbiel@gmail.com (M.P.); ewaszpyrka@interia.pl (E.S.)

**Keywords:** dental calculus, dietary fatty acids, GC-MS, archaeology, anthropology

## Abstract

Dental calculus analysis can be a valuable source of archaeological knowledge, since it preserves not only microbial and host biomolecules but also dietary and environmental debris, as well as metabolic products likely originating from dietary and craft activities. Here we described GC-MS analysis of a set of historic dental calculus samples from the front teeth of the mandibles of seven individuals found in 17th- and 18th-century graves in the city of Rzeszow, located in South-eastern Poland. We have found that only saturated fatty acids, which are characteristic for fats of animal origin, were present in the tested samples. Our preliminary results indicate that the diet of modern-period inhabitants of Rzeszow was rich in animal products, such as meat and dairy products.

## 1. Introduction

Dental plaque is an example of biofilm, a very common way for micro-organisms to exist in many environments. Bacterial biofilm is defined as a complex aggregation of micro-organisms connected with each other and attached to various surfaces, surrounded by the extracellular polymeric substance (EPS) they produce [1]. It consists of bacteria that change depending on the degree of maturity of the plaque and remain in complex relationships with one another. The molecular analysis of 16S rRNA genes, amplified from plaque samples, has identified over 200 bacterial taxa, of which approximately more than half are currently unculturable [2]. Adhesion to the surface of the hard tissues of the tooth, the vicinity of various species of bacteria, the presence of extracellular substances in the multicellular system, and heterogeneous structure and operation allow for biofilm homeostasis even under difficult environmental conditions, such as the lack or excess of substrate or the presence of bactericidal preparations [3]. Microbes metabolize carbohydrates to form carboxylic acids such as lactic, acetic, and propionic acids. These acids have the ability to demineralize the hard tissues of the tooth, resulting in tooth decay. The metabolism of plaque depends on the type, composition, and consistency of the consumed food, the frequency of eating, and the individual plaque’s acid-producing potential [4].

Dental calculus (a form of hardened dental plaque) analysis can be a valuable source of archaeological knowledge, because a mineralized oral plaque biofilm preserves biomolecules such as DNA and protein in the archaeological record over long periods of time [1,5,6,7,8,9]. Moreover, ancient dental calculus preserves not only microbial and host biomolecules but also dietary and environmental debris including pollen, starch granules, and metabolic products [6,10,11,12,13]. Therefore, the samples of ancient dental calculus allow deep-time genetic and non-genetic molecular anthropology approaches to studying changes in human behavior based on evolution of the oral biofilm and disease processes, and co-evolution of the oral microbiome and host [14]. Here we present gas chromatography-mass spectrometry (GC-MS) analysis of a set of historic dental calculus samples from the front teeth of the mandibles of seven individuals found in graves discovered in 2017 in the city of Rzeszow.

## 2. Materials and Methods

### 2.1. The Material and Its General Context

Rzeszow is the capital of the Subcarpathian region located in South-eastern Poland. The reconstruction of one of the oldest and most important streets of this city was a unique opportunity to enrich knowledge about its history through archaeological research. Excavations were carried out in the period from January to April 2017 by the Foundation for Rzeszow Archaeological Centre (in Polish *Fundacja Rzeszowskiego Ośrodka Archeologicznego*), at site number 17. As expected, the research brought an abundance of findings, including two church cemeteries. One of them was established next to the parish church (St. Wojciech and St. Stanisław Church, Farny Square). Its southern part was uncovered, with 39 burials located on four levels: 110–140 cm; 140–160 cm; 160–180 cm, and 180–220 cm [15]. The anthropological examination of the bones discovered in part of the cemetery with 39 graves showed that they contained the remains of up to 72 people [15]. This number was obtained assuming that some bones from different graves could not be connected to a specific skeleton. It should be noted that present bone remains belonged probably to people from the lower social classes. The above conclusion was made on the base localization of burials on the edge of the cemetery, near to the wall, where poor people (including children) were usually buried. Additionally, the discovered skeletons were buried mostly without equipment or coffins [16,17]. The dating of discovered burials is based on the movable antique material. This corresponds to the concrete radiocarbon tests of samples taken from the skeletons at different levels. Most of the burials are dated back to the 17th century, some probably to the beginning of the 18th century [16,17]. Nearby, at the Church of the Holy Cross, three burials were discovered—two men and one adult of unknown sex. Piarist monks or people associated with Collegium Resoviense were presumably buried here in the period from the 17th to the beginning of the 18th century [16]. The exposed human bone remains were explored and submitted for anthropological analysis at the Institute of Archaeology of the University of Rzeszow. The state of preservation of the skeletons was various, more or less incomplete; some were without skulls or teeth. Children’s burials dominated in the examined part of the cemetery—21 individuals were found in total. In some graves, the bones of other individuals were additionally found and marked with consecutive numbers within a given burial [15,18].

While at the parish church, the teeth of adults were preserved in 16 cases: 6—maxilla and mandible, calculus; 4—maxilla and mandible, few teeth present, no calculus; 2—maxilla, no calculus; 1—maxilla, no teeth, complete alveolar obliteration; 3—mandible, a trace of calculus. At the Church of the Holy Cross, only the skeleton number 1 was preserved with maxillary and mandibular teeth and calculus; the other two skeletons were without skulls and teeth.

**Figure 1:**
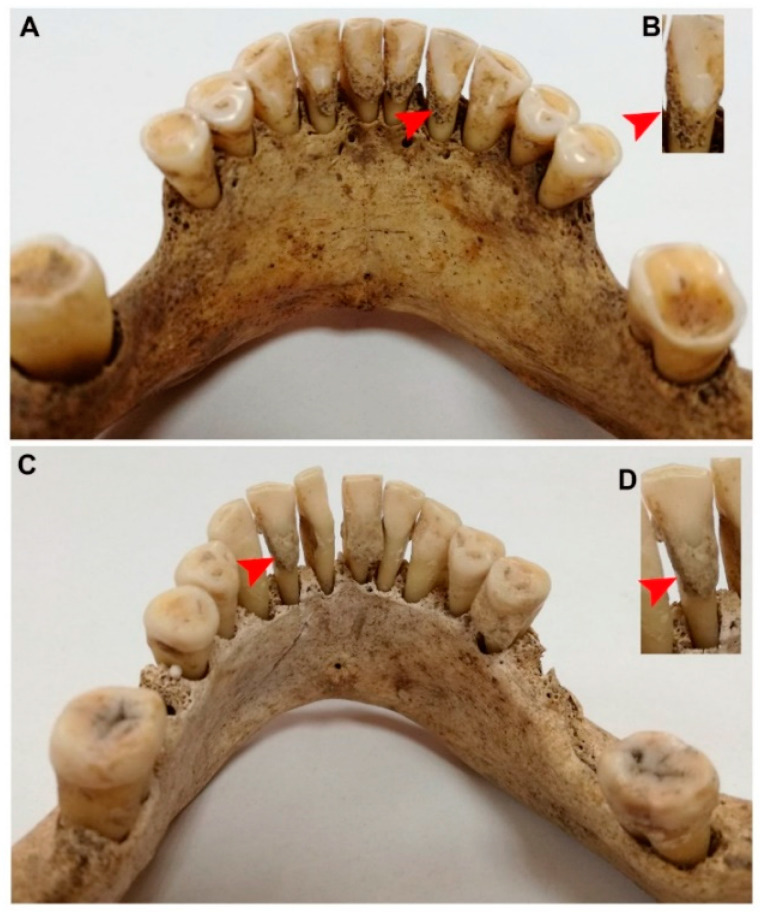
Mandibles with teeth and dental calculus collected from two adults of 17th-century inhabitants of Subcarpathian region: (**A**) mandible, grave 13, male, *maturus*; Farny Square, Rzeszow, site 17; (**B**) magnification of right second incisor with dental calculus; (**C**) mandible, grave 3, female, *maturus–senilis*; Farny Square, Rzeszow, site 17; (**D**) magnification of left second incisor with dental calculus. Red arrowhead indicates dental calculus.

The dental calculus samples were collected from adults (4 women and 3 men), 6 of whom were buried near the parish churchyard: grave 2 (individual 2), grave 3, grave 12, grave 13, grave 23, grave 35 (individual 1) and one near the Church of the Holy Cross: grave 1. The samples were obtained from all the mandibles with dentition and calculus. The dental calculus samples were collected from incisors of each person chosen for the analysis (Figure 1).

### 2.2. Analytical Methods

The analysis and samples were prepared according to the publication [14] with the following modification. All seven historic dental calculus samples were pulverizationed with a stainless steel pestle and then 15 mg was decalcified with 100 µL of 4% formic acid (Chempur, Piekary Slaskie, Poland) at 4 °C for 12 days. Next, 75 µL of 1 M ammonium hydroxide (Chempur, Piekary Slaskie, Poland) was added, then samples were extracted with 350 µL CH_3_OH (Honeywell Specialty Chemicals Seelze GmbH, Hannover, Germany) + 350 µL acetonitrile (Chempur, Piekary Slaskie, Poland) (2:2:1 methanol:acetonitrile:water). For GC-MS analysis, dried extract was derivatized for 90 min with 20 mg/mL methoxyamine hydrochloride (J & K Scientific, Beijing, China) in pyridine (Chempur, Piekary Slaskie Poland) at 20 °C (10 µL) and then with MSTFA (TCI, Tokyo, Japan) for 30 min at 37 °C (10 µL). Samples were analyzed by GC-MS/MS; 1 µL of sample was injected onto a HP-5MS column. Temperature was held at 50 °C for 1 min, then ramped to 320 °C at a rate of 11 °C/min, then held at 320 °C for 4.40 min. Molecules were analyzed with electron ionization (EI) and full scan mode of 50–650 m/z range.

The extracts were determined on a gas chromatograph: model 7890A (Agilent Technologies, Palo Alto, CA, USA) equipped with a model 7000 triple quadrupole mass detector (QQQ), autosampler, and HP-5 MS Ultra Inert/30 m × 0.25 mm I.D. × 0.25 µm. The spectra obtained were also compared with the NIST database (The NIST Mass Spectral Search Program, Mass Spectral Library Ver. 2.2, Gaithersburg, MD, USA) and similarity values higher than 85% were applied. The Agilent Technologies Mass Hunter version 07.06 (Palo Alto, CA, USA) was used as software for acquisition and quantification of the analysis data. For relative quantification of results, normalization method was applied [19]. Recovery during trimethylsilyl (TMS) derivatization step is from 90% to 106%, and precision %RSD is less than 4% [20].

We have validated the method for fatty acid methyl ester (FAME) because of the commercial availability of analytical standard FAMEs mixture (37 Supelco Component FAME MIX, Sigma Aldrich, Merck, Germany). The analytical performance was very good in terms of linearity, limits of quantifications (LOQs), and precision expressed as repeatability. LOQs for each fatty acid were calculated as a signal-to-noise ratio (S/N) of 10:1 and were in the range of 1–3 mg/kg for individual fatty acids. The linearity for each FAME was determined on the basis of 5-point calibration curves and was in the range 0.972–1. The recovery analysis was done in five repetitions for samples with addition of triglyceride internal standard (ISTD, tripentadecanoin) and was equal to 74%. The precision was expressed as relative standard deviations obtained in the recovery studies for ISTD, and it was 6%.

## 3. Results and Discussion

We identified a total of four major fractions of putative dietary fatty acids and/or their derivatives in the archaeological samples (Table 1).

**Figure 2:**
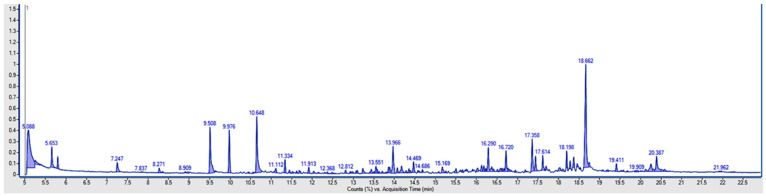
The chromatogram of dental calculus sample (retention times for tetradecanoic acid—16.720 min, pentadecanoic acid—17.358 min, hexadecanoic acid—18.662 min, and octadecanoic acid—20.387 min).

No significant differences in the content of individual fatty acids by sex or age were detected (Table 1). However, only saturated fatty acids were found in the tested samples, which are characteristic for fats of animal origin. The remaining acids were below the limit of quantification of the method (>3 mg/kg). Moreover, the lack of monounsaturated (MUFAs) and polyunsaturated fatty acids (PUFAs) in historic calculus may be partially explained by the decreased oxidative stability of fatty acids with decreasing saturation [14]. Velsko et al. compared the dental calculus metabolome in modern and historic samples. In all modern dental calculus samples, two PUFAs, mead acid (20:3n9) and dihomo-linolenate (20:3n3 or n6), were identified, but were not observed in historic samples [14]. Overall, we found a low proportion of putative dietary tetradecanoic acid when compared with other detected fatty acids (Table 1, Figure 2).

Tetradecanoic acid is also known as myristic acid (MA) and is a member of the class of compounds belonging to long-chain saturated fatty acids. It can be found in a number of food items such as vegetables and fruits [21]. MA has been found only in small amounts (<1% of total free fatty acids, FFAs) in animal tissue, but it is highly abundant in milk fat (7–12%) [22]. Pentadecanoic acid and octadecanoic acid were detected in all samples at the same low level. Octadecanoic acid, known as stearic acid, is one of the most common saturated fatty acids. Animal and plant fats contain stearic acid as a glycerol ester; however, stearic acid is more abundant in animal fat (up to 30%) than in vegetable fat (typically < 5%) [23]. Pentadecanoic acid—a saturated fatty acid—is one of the most common, comprising 1.2% of cow milk fat [24]. The most abundant saturated fatty acid fraction detected in historic dental calculus samples was hexadecanoic acid (known as palmitic acid) (37.9–65.9%)—the most abundant saturated fatty acid in nature. Palmitic acid is the most common saturated fatty acid in animal lipids and plants, where it occurs as a glycerol ester [25]. It is the main component of fresh red and white meat such as lamb, rabbit thigh, pork loin, and chicken. Moreover, chicken, duck, goose, and turkey egg yolk are abundant in hexadecanoic acid [25,26].

The fatty acids such as tetradecanoic, pentadecanoic, hexadecanoic, and octadecanoic detected in the present study were identified also by Velsko et al. who used the GC-MS method to analyze historic calculus (originated from skeletons from a UK cemetery used from 1770–c.1855) [14]. They have also found one other saturated fatty acid like heptadecanoic acid, two MUFAs (palmitoleic and oleic acids), and one PUFA (arachidic acid) [14]. These analyses were based only on qualitative and not quantitative measurement [14]. Gismondi et al. investigated dietary and medicinal habits of the medieval population of Santa Severa (Rome, Italy) from the 7th–15th centuries. Generally, MUFAs (e.g., docosenoic and octadecenoic acids), PUFAs (e.g., octadecadienoic and octadecatrienoic acids), and ω3-fats (e.g., eicosapentaenoic and docosahexaenoic acids) were the most recurrent molecules detected. This fatty acids profile suggests a diet typical for a coastal Mediterranean population based on plant foods, such as seeds, vegetables, fruits, and ω3-fatty acids which could be associated with the ingestion of plant and/or aquatic resources (e.g., seaweeds, mollusks, and blue fishes). The authors also did not provide amounts for detected compounds. Each chemical compound was identified by comparison of its mass spectrum with those registered in the NIST Library 14 with similarity values higher than 85% [27].

The obtained results do not exclude the presence of other foods in the diet of the in-habitants of Rzeszow but indicate that the presence of animal foods in human nutrition was greater than expected. The historic research has provided data that the diet of residents of 18th-century England contains a large portion of meat [28]. Similar conclusions were provided by other authors that are claiming that the poorer families mostly in the late 17th and early 18th century in England had often kept animals that could provide food for them, such as cows, pigs, and geese [29]. It is interesting that, at the same time, poor English people in the 18th century had limited access to fruit and only a very select, minuscule group of wealthy people had access to fruit [29]. The above-mentioned historical notes can partially strengthen our hypothesis. Unfortunately, there are still too little data in the literature, especially about bioarchaeological analyses of samples from the 17th to the beginning of the 18th century in Poland. Therefore, one should be careful with the proposed archaeological hypothesis, which should be verified by other historic sources.

## 4. Conclusions

In summary, our preliminary results can indicate that the diet of inhabitants of Rzeszow in the modern period was rich in animal products such as meat and dairy. The detected proportion of fatty acid profiles also can indicate a low proportion of plant foods. The obtained bioarchaeological results are the first of their kind for the Subcarpathian region. The presented results are among the bioarchaeological data known so far concerning the analysis of historic dental calculus of the 17th- and 18th-century inhabitants of the Subcarpathian region.

## Figures and Tables

**Table 1:** Fatty acids detected in the analyzed dental calculus samples.

	Sample 1	Sample 2	Sample 3	Sample 4	Sample 5	Sample 6	Sample 7
**Sex**	**F**	**F**	**F**	**M**	**F**	**M**	**M**
**Age**	*maturus*	*maturus–senilis*	*adultus*	*maturus*	*adultus*	*adultus*	*maturus*
**Grave no.**	2 ind. 2	3	12	13	23	35 ind. 1	1, HC
**Fatty Acid %**							
Tetradecanoic acid	19.7	13.0	−	−	6.6	10.3	−
Pentadecanoic acid	24.2	13.0	23.5	11.1	12.3	31.1	11.6
Hexadecanoic acid	48.2	56.6	53.0	61.1	65.9	37.9	58.1
Octadecanoic acid	7.9	17.4	23.5	27.8	15.2	20.7	30.3

F—female, M—male, HC—the Church of the Holy Cross.

## Data Availability

The data presented in this study are available on request from the corresponding author.

## Sample Availability

Samples of the historic dental calculus are not available from the authors.
